# Twist1-positive epithelial cells retain adhesive and proliferative capacity throughout dissemination

**DOI:** 10.1242/bio.019703

**Published:** 2016-07-11

**Authors:** Eliah R. Shamir, Kester Coutinho, Dan Georgess, Manfred Auer, Andrew J. Ewald

**Affiliations:** 1 Departments of Cell Biology and Oncology, Center for Cell Dynamics, Johns Hopkins University School of Medicine, 855 N. Wolfe St, Baltimore, MD 21205, USA; 2 Molecular Biophysics and Integrated Bioimaging Division, Lawrence Berkeley National Laboratory, 1 Cyclotron Road, MS Donner, Berkeley, CA 94720, USA

**Keywords:** Twist1, Dissemination, Epithelial-mesenchymal transition, Cell migration, Intercellular junctions, Proteolysis

## Abstract

Dissemination is the process by which cells detach and migrate away from a multicellular tissue. The epithelial-to-mesenchymal transition (EMT) conceptualizes dissemination in a stepwise fashion, with downregulation of *E-cadherin* leading to loss of intercellular junctions, induction of motility, and then escape from the epithelium. This gain of migratory activity is proposed to be mutually exclusive with proliferation. We previously developed a dissemination assay based on inducible expression of the transcription factor *Twist1* and here utilize it to characterize the timing and dynamics of intercellular adhesion, proliferation and migration during dissemination. Surprisingly, Twist1^+^ epithelium displayed extensive intercellular junctions, and Twist1^–^ luminal epithelial cells could still adhere to disseminating Twist1^+^ cells. Although proteolysis and proliferation were both observed throughout dissemination, neither was absolutely required. Finally, Twist1^+^ cells exhibited a hybrid migration mode; their morphology and nuclear deformation were characteristic of amoeboid cells, whereas their dynamic protrusive activity, pericellular proteolysis and migration speeds were more typical of mesenchymal cells. Our data reveal that epithelial cells can disseminate while retaining competence to adhere and proliferate.

## Abstract (precis)

**Summary:** Twist1^+^ epithelium retains intercellular junctions and proliferative capacity. During dissemination, Twist1^+^ cells retain adhesive capacity and exhibit features of both amoeboid and mesenchymal migration modes.

## INTRODUCTION

Dissemination, the release of cells and their migration away from epithelial tissues, plays an essential role in both normal development and cancer metastasis. Cells adopt diverse and plastic migration modes to invade into and migrate through the surrounding 3D extracellular matrix (ECM) as single cells or as collective groups (Friedl and Wolf, 2010; Sanz-Moreno et al., 2008). Single-cell dissemination inherently involves release of cell-cell interactions and gain of cell-matrix interactions in order to escape the epithelium and enter the surrounding ECM (Clark and Vignjevic, 2015; Friedl and Wolf, 2010). In cancer, loss of cell-cell adhesion can occur directly or can be a component of a broader program of epithelial-to-mesenchymal transition (EMT) (Bogenrieder and Herlyn, 2003; Kalluri and Weinberg, 2009).

The EMT model proposes that epithelial tumor cells convert to mesenchymal cells to acquire migratory capacity (Lamouille et al., 2014; Nieto, 2013). This concept derives from study of normal development and has been proposed to explain metastasis, supported by the expression of EMT transcription factors in invasive epithelial cancers (Blanco et al., 2002; Martin et al., 2005; Tsai et al., 2012; Yang et al., 2004; Yang and Weinberg, 2008). In this model, dissemination is induced by *E-cadherin* repression, leading to cell-cell junction disassembly, loss of apicobasal polarity and detachment from basement membrane anchoring (Lamouille et al., 2014; Peinado et al., 2007; Thiery, 2002; Thiery et al., 2009). The global loss of epithelial differentiation is thought to directly lead to delamination of protrusive, elongated cells that employ a mesenchymal strategy of migration (Lamouille et al., 2014). EMT has been a dominant conceptual framework for epithelial dissemination. However, it has been difficult to demonstrate the entire process in a single experimental system.

We recently demonstrated the sufficiency of the EMT transcription factor Twist1 to induce single-cell dissemination from mouse mammary organoids cultured within a 3D laminin-rich ECM (Matrigel) (Shamir et al., 2014). Dissemination was not associated with loss of epithelial gene expression and required E-cadherin, counter to the EMT model (Shamir et al., 2014). In the present study, we leveraged our Twist1 assay to define how single-cell dissemination is accomplished at the cellular level. We use a combination of fluorescent reporters, time-lapse DIC and confocal imaging, small molecule inhibitors and transmission electron microscopy (TEM) to track Twist1^+^ cell behaviors and ultrastructure throughout dissemination. We demonstrate that Twist1^+^ cells disseminate despite cell-cell junctions, remain capable of adhesion and proliferation throughout dissemination, and migrate in a hybrid fashion, with characteristics of both mesenchymal and amoeboid modes.

## RESULTS

### Junctional complexes connect cells within Twist1^+^ epithelium

**Fig. 1.**
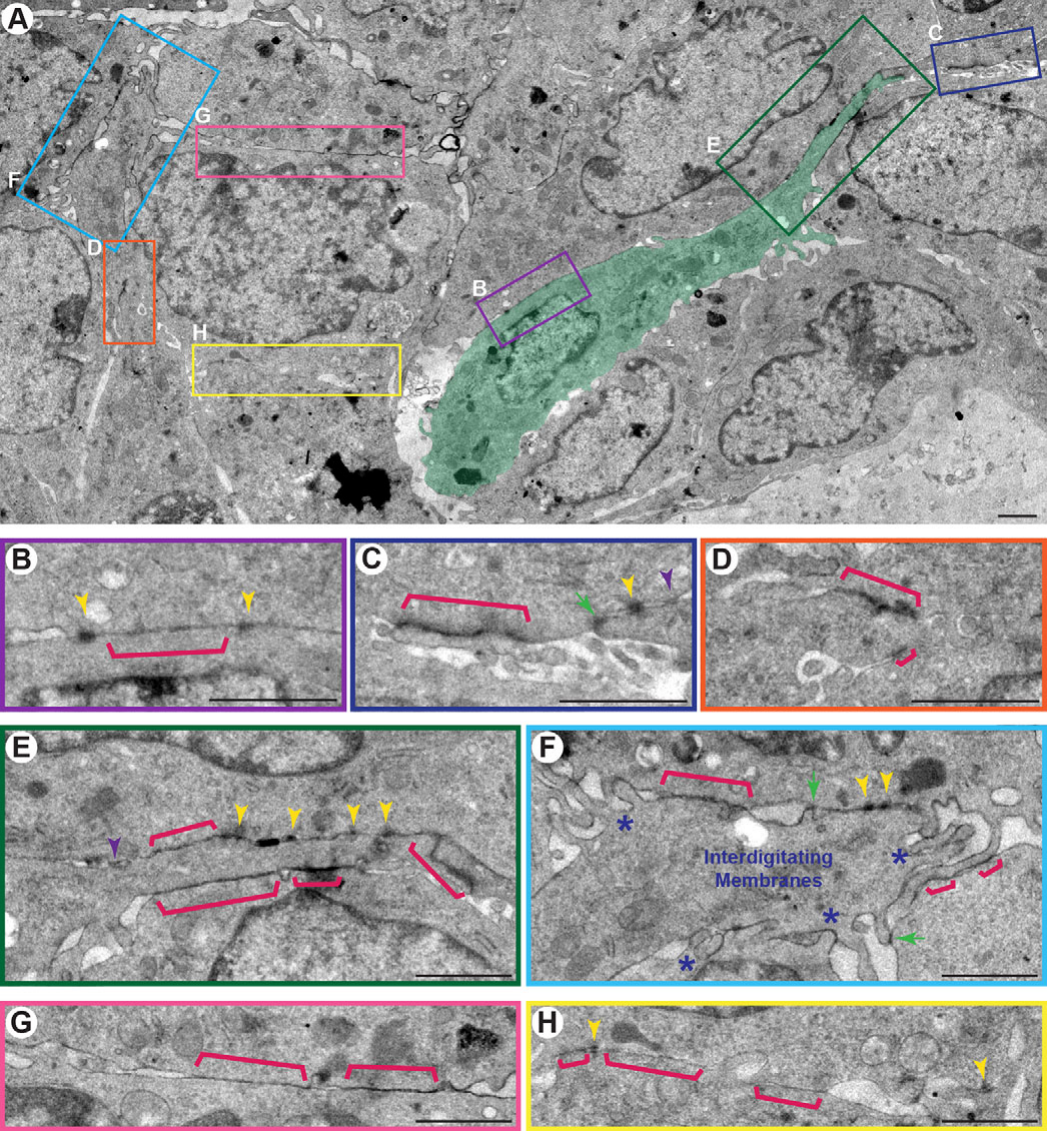
**Multiple classes of junctions connect cells within Twist1^+^ epithelium.** TEM was used to define the ultrastructure within the multilayered epithelium of *CMV::rtTA;TRE-Twist1* organoids. (A) Interior epithelial cells away from the basal tissue surface were unpolarized and frequently tightly packed. Individual cells could appear migratory (green pseudocolor). (B-H) Junctions were classified into four morphologically distinct categories. Bar junctions (B-H, pink brackets) were the most commonly observed class, localized electron density to the membrane, and lacked intercellular gaps. Darkly staining punctate junctions (B,C,E,F,H, yellow arrowheads) accumulated electron density in the adjoining cytoplasm, and sandwich junctions (C,E, purple arrowheads) localized electron density to the membrane and contained an intercellular, electron lucid space. Cells were also connected by lateral interdigitating membrane protrusions (F, blue asterisks) and contact junctions (C,F, green arrows) between protrusions and cell membranes. Scale bars: 1 μm. All TEM images are from high-pressure frozen, freeze-substituted samples that were pre-fixed with 3% glutaraldehyde and stained with Ruthenium Red.

Constitutive *Twist1* expression disrupts polarized tissue architecture at the light microscopy level (Shamir et al., 2014). However, light microscopy cannot resolve intercellular junctions, and so we first sought to use TEM to define the ultrastructural adhesive environment inside epithelium ubiquitously expressing *Twist1* compared to normal epithelium (organoids isolated from *CMV::rtTA;TRE-Twist1* mice grown with and without doxycycline) (Fig. 1) (TRE, tetracycline responsive element). The expectation from the EMT model was that cell-cell adhesion in Twist1^+^ epithelium would be disrupted and that cells would be loosely connected with few or no detectable junctions. To test this prediction, we quantified junctions in both Twist1^+^ and control epithelium. The observed junctions did not correspond exactly to classical junctions from simple epithelia, and so we defined four morphologically distinct categories: bar, punctate, sandwich, and contact junctions (defined in Materials and methods and in Fig. S1). Surprisingly, we observed an increase in the average total number of junctions per cell in Twist1^+^ epithelium (21 junctions) compared to control epithelium (16 junctions; *P=0.02; 30 cells per condition).

The membranes of adjacent Twist1^+^ cells were tightly apposed (Fig. 1A-H) and interspersed with punctate, electron-dense junctions (Fig. 1B-F,H, yellow arrowheads). The punctate junctions localized electron density at the membrane and in the cytoplasm and displayed a varied accumulation of intermediate filaments (Fig. 1B-F,H, yellow arrowheads). Their appearance is most consistent with desmosomes, though we cannot exclude that they may have mixed molecular architecture. We also observed junctions with electron density localized to the membrane without detectable intercellular space (bar junctions; Fig. 1B-H, pink brackets). In thin sections, these junctional connections could appear continuous or intermittent, at distinct foci along the cell-cell interface. The appearance of bar junctions is most suggestive of tight junctions (TJs). An additional morphological class of junctions accumulated electron density to the membrane but not the cytoplasm and had a detectable intercellular, electron-lucid gap (sandwich junctions; Fig. 1C,E, purple arrowheads). In regions with more extensive intercellular space, cells were observed to have interdigitating membrane protrusions (Fig. 1F, blue asterisks). These membrane protrusions were observed to make junctions with the membranes of adjacent cells (contact junctions; Fig. 1C,F, green arrows). Apparently migratory, elongated interior cells (Fig. 1A, cell pseudocolored green) also maintained junctional connections to neighboring cells, both at lateral cell-cell borders (Fig. 1B) and at the cell front (Fig. 1E). Punctate and contact junctions were significantly enriched in Twist1^+^ versus control epithelium (Fig. S1). These data reveal that *Twist1*-expressing epithelial cells retain multiple classes of intercellular junctions, though further immuno-EM will be required to distinguish their molecular composition.

### Internal Twist1^+^ cells can migrate to the tissue surface

Given the maintenance of junctional adhesion within Twist1^+^ epithelium, it was possible that all successfully disseminating Twist1^+^ cells start out at the tissue-ECM surface. We therefore sought to track the fate of interior Twist1^+^ cells. We leveraged a genetic mosaic model of *Twist1* expression in which a Cre-inducible rtTA (*R26::LSL-rtTA*; Belteki et al., 2005) (LSL, Lox-Stop-Lox) and a fluorescent Cre biosensor (*mT/mG*; Muzumdar et al., 2007) allows us to distinguish Twist1^+^ (green) and Twist1^–^ (red) cells in real-time, throughout dissemination. We induced rare, mosaic activation of *Twist1* and monitored Twist1^+^ interior cells by confocal microscopy. Mammary epithelium consists of inner luminal epithelial cells and basally positioned myoepithelial cells, and the two cell types typically remain within their respective layers. In contrast, we observed that interior Twist1^+^ cells migrated from luminal cell layers into the basal cell layer (Fig. S2A,A′). Cells initiated protrusions basally while maintaining the main cell body within the internal layer (Fig. S2A,A′). As the protrusive front widened, the cell volume redistributed across the length of the luminal layer. Finally, rear retraction completed translocation to the basal layer (Fig. S2A,A′). In a similar squeezing fashion, interior Twist1^+^ cells were also observed to protrude past the basal cell layer and migrate directly to the tissue surface in a single step (Fig. S2B-B″). Basally positioned and actively disseminating cells both exhibited nuclear-localized Twist1 immunoreactivity (Fig. S2C-C″, blue arrowheads). We conclude that Twist1^+^ cells do not obey normal cell type-specific or tissue boundaries and can migrate past Twist1^–^ cells to the tissue-ECM border.

### Twist1^+^ cells protrude into the matrix while retaining junctions

**Fig. 2.**
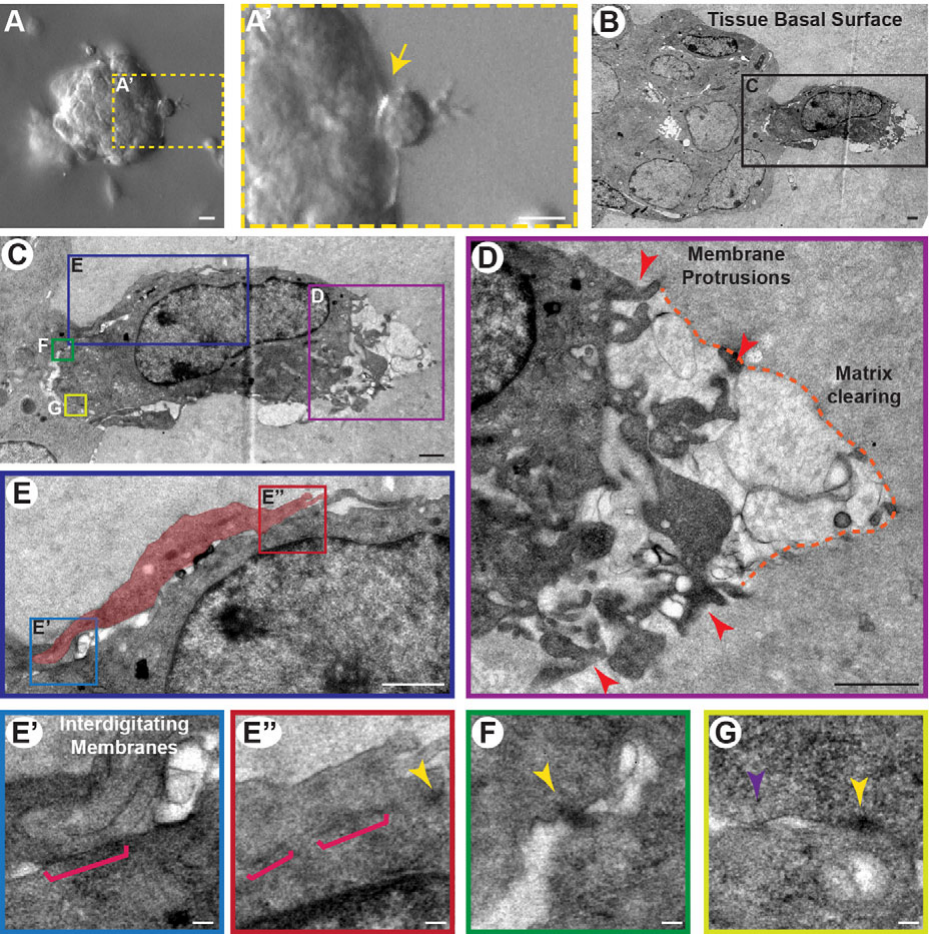
**Twist1^+^ cells protrude into the matrix despite junctions at the rear.** The junctional environment in basally positioned Twist1^+^ cells was analyzed in *CMV::rtTA;TRE-Twist1* organoids. (A) Representative DIC image of a *Twist1*-expressing organoid showing a cell that protrudes into the ECM while still attached to the epithelium (A′). Yellow arrow in A′ indicates the cell-cell interface of interest. Scale bars: 10 μm. (B,C) TEM was used to examine the adhesive interactions of cells on the tissue basal surface. Scale bars: 1 μm. (D) Sites of membrane protrusion (red arrowheads) were observed at the cell front and often corresponded with matrix clearing (orange dashed line). Scale bar: 1 μm. (E-G) Protrusive cells were connected to cells at the side and/or rear by at least one junction (11 regions of protrusive activity across five Twist1^+^ organoids). Red pseudocolor in E indicates a neighboring cell wrapped around the protrusive cell. Scale bar: 1 μm. Pink brackets in E′-E″ delineate bar junctions; yellow arrowheads in E″-G indicate punctate junctions; purple arrowhead in G indicates a sandwich junction. Scale bars: 0.1 μm. All TEM images are from high-pressure frozen, freeze-substituted samples that were pre-fixed with 3% glutaraldehyde and stained with Ruthenium Red.

Having demonstrated by TEM that interior Twist1^+^ cells maintained junctional connections, we next sought to examine the adhesive interactions of Twist1^+^ cells at the tissue-ECM interface. We examined organoids ubiquitously expressing *Twist1* for rare events that captured basally positioned cells fixed during the process of dissemination (Fig. 2A,A′,B). We observed cells on the tissue surface with extensive, ECM-directed membrane protrusions (Fig. 2C,D, red arrowheads). In the area proximate to these protrusions, we observed a decrease in electron density relative to the surrounding ECM, which we inferred to be proteolytic matrix clearing (Fig. 2D, orange dashed line). These cells were connected to neighboring cells by multiple junctions at both rear and lateral cell surfaces (Fig. 2E-G). We identified 11 regions across five organoids where either a single cell on the tissue surface or a cell within an invasive group displayed protrusive activity. In all 11 examples, at least one small junction was present at the interface between the protrusive cell and an adjoining cell. Due to the challenge in detecting these junctions in a thin section by TEM, our ability to resolve their identity was limited. However, their morphology was consistent with the bar junctions (Fig. 2E′,E″, pink brackets), punctate junctions (Fig. 2E″,F,G, yellow arrowheads), and sandwich junctions (Fig. 2G, purple arrowhead) observed connecting interior Twist1^+^ cells. These data show that disseminating Twist1^+^ cells simultaneously protrude into the ECM at the front and retain junctions with cells in the epithelium.

### Disseminating Twist1^+^ cells remain adherent to normal epithelial cells

**Fig. 3.**
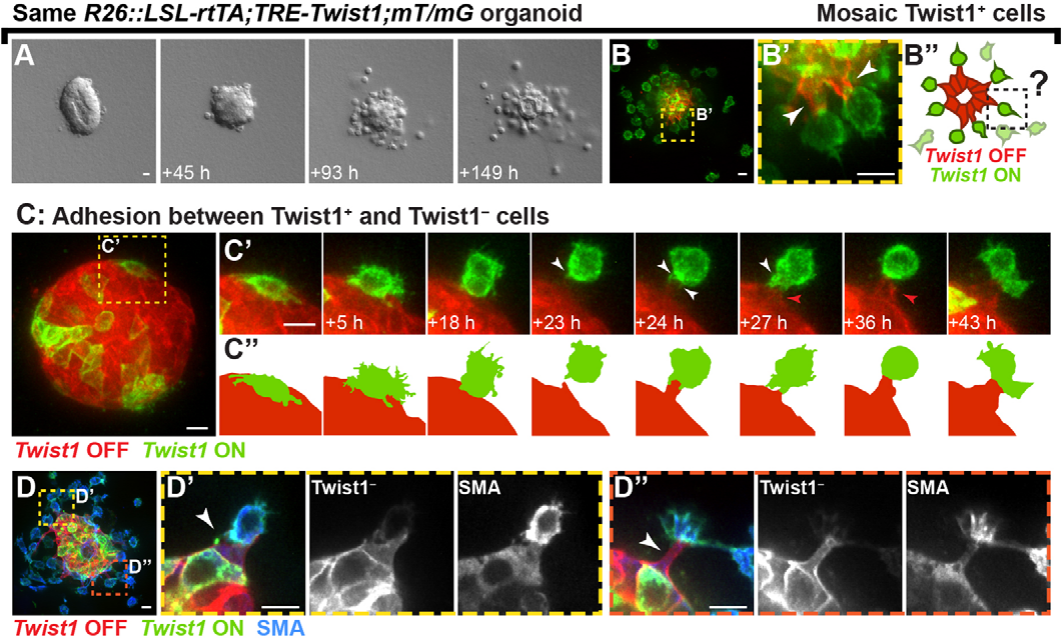
**Twist1^+^ and Twist1^–^ cells can adhere to each other during dissemination.** Adeno-CMV-Cre was used to induce genetic mosaic activation of *Twist1* in *R26::LSL-rtTA;TRE-Twist1;mT/mG* organoids. Organoids were cultured in basal medium without FGF2, and the *mT/mG* biosensor served as an indirect reporter of rtTA^+^Twist1^+^ cells (green). (A,B) Organoids with a high percentage of Twist1^+^ cells disseminated single cells but retained a cystic core composed of Twist1^–^ cells (red). Twist1^–^ cells that maintained contact with Twist1^+^ cells were stretched basally into the ECM (white arrowheads) (B′,B″). (C) Confocal time-lapse microscopy was used to observe interactions between Twist1^+^ and Twist1^–^ cells. Twist1^+^ cells that had just detached from the epithelium initiated rear protrusions (white arrowheads) and reestablished contact with a Twist1^–^ cell (red arrowheads) (C′-C″). This adhesion often resulted in retention of the Twist1^+^ cell (*n=*35 cells in 18 organoids imaged by time-lapse across three biological replicates). (D) Basally stretched Twist1^–^ cells (white arrowheads) were negative for the myoepithelial marker smooth muscle actin (SMA). Cropped images in D′ and D″ are rotated for clarity. Scale bars: 10 μm.

Conventional models of single-cell migration are characterized not only by a lack of cell-cell junctions but also by decreased cell-cell contact with other epithelial cells (Clark and Vignjevic, 2015; Friedl and Wolf, 2010). This framework would predict that Twist1^+^ cells, once detached from the epithelium, would infrequently contact and lack an ability to adhere to other cells. We used time-lapse microscopy to monitor for epithelial cell-cell interactions during dissemination in genetic mosaic organoids. We first induced *Twist1* in a high fraction of cells and observed extensive dissemination, with organoids shedding single cells but retaining a residual Twist1^–^ epithelial core (Fig. 3A,B-B″). Interestingly, we consistently detected Twist1^–^ cells that were stretched basally into the ECM as they maintained contact with protrusive Twist1^+^ cells (Fig. 3B′,B″). We next used confocal microscopy to better understand the real-time dynamics between these two cell populations. We observed that Twist1^+^ cells on the tissue surface frequently migrated away from the epithelium while maintaining dynamic contact with Twist1^–^ cells at the rear, seemingly unable to fully detach (Fig. 3C-C″, white arrowheads). These intercellular contacts resulted in basal extension of the Twist1^–^ cell out of the organoid (Fig. 3C′,C″, red arrowheads). Disseminated Twist1^+^ cells that reestablished a cell-cell border with the Twist1^–^ cell typically rounded up and became less protrusive (Fig. 3C′,C″). Moreover, this heterotypic adhesion frequently resulted in retention of Twist1^+^ cells and a failure to fully detach. Immunofluorescence demonstrated that basally stretched Twist1^–^ cells were negative for the myoepithelial cell marker smooth muscle actin (SMA) and were thus presumably luminal epithelial cells (Fig. 3D-D″). We conclude that, contrary to expectation, Twist1^+^ cells and normal, Twist1^–^ epithelial cells retain the ability to adhere to each other throughout dissemination.

### Twist1^+^ cell release and migration occur by amoeboid motility

**Fig. 4.**
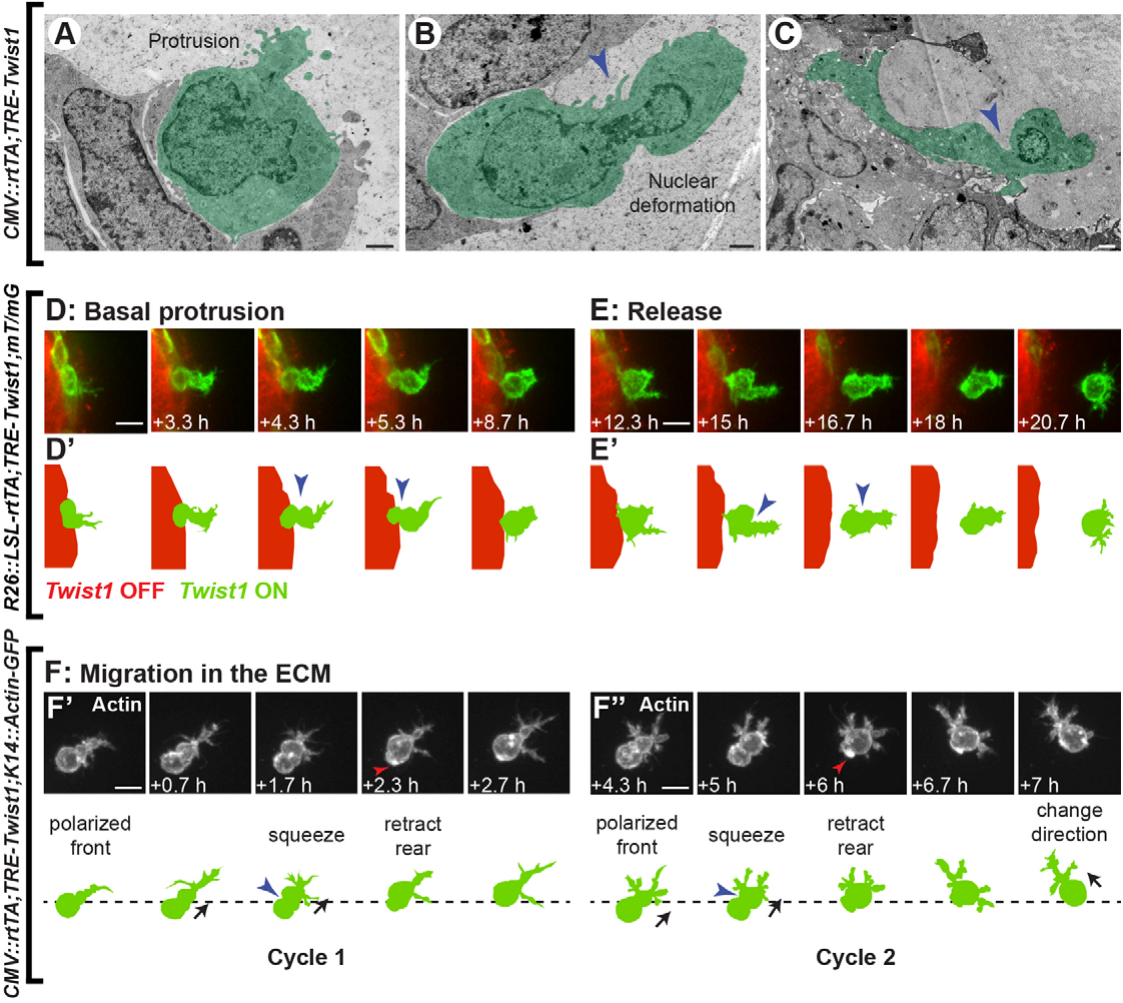
**Twist1^+^ cells display amoeboid morphology during release and migration.** (A-C) TEM was used to examine the morphology of Twist1^+^ cells during dissemination in *CMV::rtTA;TRE-Twist1* organoids. Cells maintained an amoeboid shape at progressive steps of protrusion into the ECM and migration out of the basal cell layer. Nuclear deformation was observed during translocation (B). All TEM images are from high-pressure frozen, freeze-substituted samples that were pre-fixed with 3% glutaraldehyde. The sample shown in panel (C) was stained with Ruthenium Red. Scale bars: 1 μm. (D-E) A low titer of Adeno-CMV-Cre was used to induce rare genetic mosaic activation of *Twist1* in isolated *R26::LSL-rtTA;TRE-Twist1;mT/mG* organoids. Dissemination was monitored by confocal time-lapse microscopy. Twist1^+^ cells translocated a rounded cell body in a squeezing fashion during migration out of the basal layer (D,D′, *n=*44 cells in 25 organoids imaged by time-lapse across three biological replicates) and detachment (E-E′, *n=*17 cells in 13 organoids imaged by time-lapse across three biological replicates). Scale bars: 10 μm. (F) The *K14::Actin-GFP* reporter was used in *CMV::rtTA;TRE-Twist1* epithelium to visualize actin dynamics during migration in the ECM, specifically in K14+myoepithelial cells. Cells repeated cycles of leading edge protrusion, cell body deformation, and rear retraction, with actin accumulation at the rear during the final step (F′,F″, red arrowheads). Black arrows indicate the direction of cell migration. Scale bars: 10 μm. Blue arrowheads in (B-F″) indicate points of cortical constriction.

Mesenchymal migration is characterized by an elongated cell body and requires high cell-matrix adhesion and extracellular proteolysis (Friedl and Wolf, 2010). In contrast, amoeboid migration is characterized by a round cell body and uses actomyosin contractility to push the rigid nucleus through ECM gaps, with minimal proteolysis (Clark and Vignjevic, 2015; Friedl and Wolf, 2010; Lämmermann and Sixt, 2009; Sahai, 2007). To define the mode of Twist1^+^ cell migration, we examined cell shape changes during cell release from the epithelium and migration in the ECM. We detected cells at progressive steps of dissemination from the basal epithelial layer by TEM (Fig. 4A-C). We observed cells within the epithelium, extending filopodia into the matrix (Fig. 4A); cells in the process of squeezing out of the epithelium, with prominent nuclear deformation (Fig. 4B, blue arrowhead); and cells with the nucleus located outside of the epithelium (Fig. 4C). We next used time-lapse microscopy to observe how such cellular shape changes accomplish cell release. Twist1^+^ cells in the basal epithelial layer first extended protrusions into the ECM perpendicular to the plane of the epithelium (Fig. 4D,D′). As these forward protrusions lengthened, the main cell body squeezed out of the basal layer of the epithelium to the tissue surface. The cell next elaborated forward protrusions, compressed its rear, and fully detached from the epithelium (Fig. 4E,E′). Morphologically, by TEM and fluorescent microscopy, Twist1^+^ cells maintained a round cell body and displayed prominent nuclear deformation and cortical contraction, consistent with amoeboid motility.

We next examined the migratory behavior of Twist1^+^ cells in the ECM. Here, we used the genetically encoded *K14::Actin-GFP* reporter to observe real-time actin dynamics during migration of disseminated K14+myoepithelial cells (*CMV::rtTA;TRE-Twist1;K14::Actin-GFP* mice; Fig. 4F-F″). Cells squeezed through the matrix in stereotypic cycles, with a protrusive filopodium at the front and a smooth, round cell body at the rear. During each cycle, the main cell body translocated from the rear to the front, with notable constriction in the middle (Fig. 4F′,F″, blue arrowheads). In the final step, the rear part of the cell retracted, with prominent concentration of actin (Fig. 4F′,F″, red arrowheads). These stereotyped cycles of forward protrusion, cell body deformation, and rear retraction were thus used to accomplish basal migration within the epithelium (Fig. S2A,B), cell release (Fig. 4D,E), and migration in the ECM (Fig. 4F). Taken together, we conclude that Twist1 induces a migratory program that involves amoeboid cell shape changes across every step of dissemination.

### Twist1^+^ cells migrate in the ECM with dynamic protrusions

**Fig. 5.**
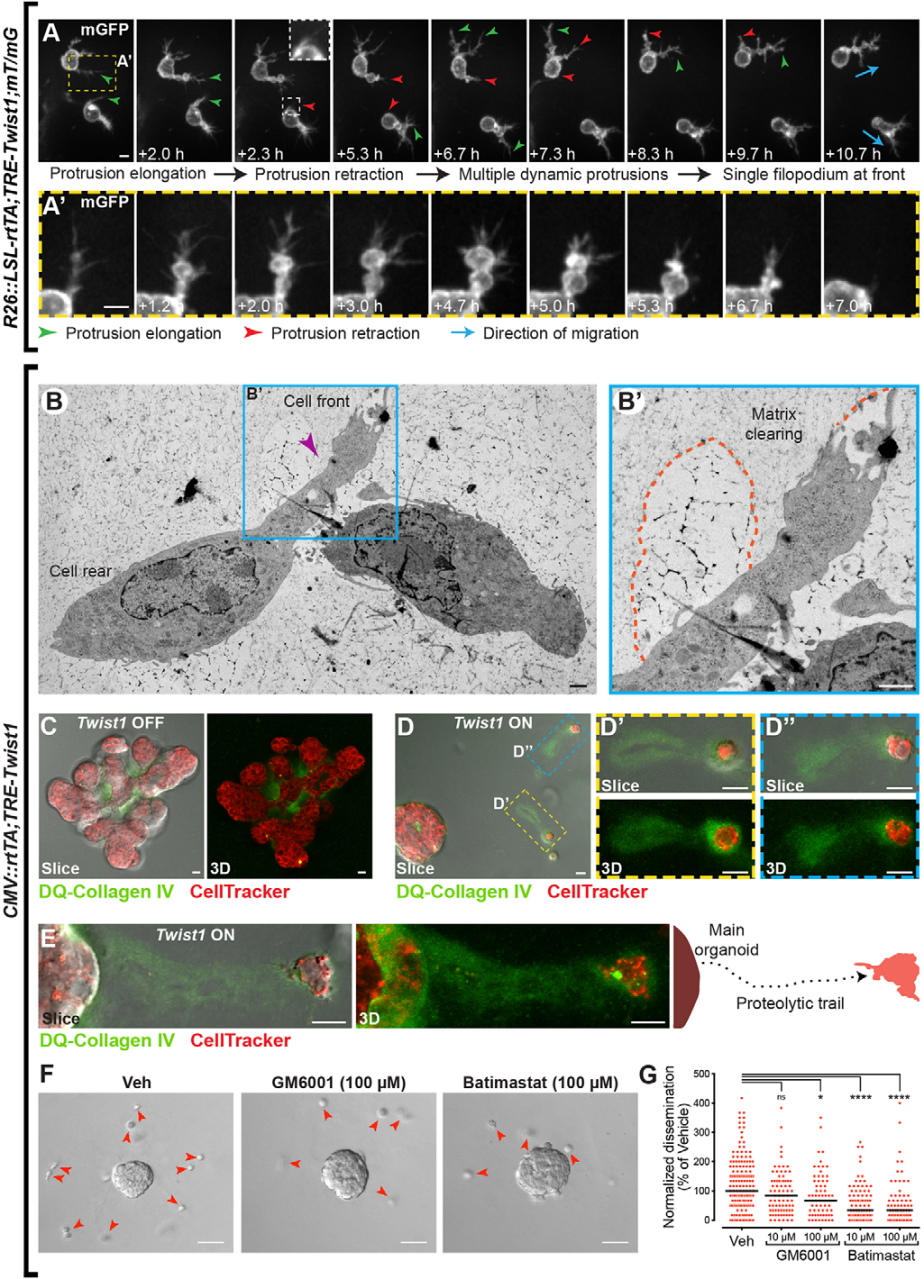
**Twist1^+^ cells migrate in the ECM with dynamic protrusions and pericellular proteolysis.** (A) Migrating Twist1^+^ cells were imaged by confocal time-lapse microscopy in isolated *R26::LSL-rtTA;TRE-Twist1;mT/mG* organoids (*n=*29 cells in 22 organoids imaged by time-lapse across three biological replicates). The membrane GFP (mGFP) fluorescence was used to visualize membrane protrusions. Twist1^+^ cells were observed to elongate (green arrowheads) and retract (red arrowheads) multiple protrusions simultaneously, with one filopodium typically persisting at the leading edge (blue arrows). Protrusions consisted of a mixture of blebs and fine, dendritic-like extensions (A′). Scale bars: 5 μm. (B) Ultrastructural examination of disseminated cells in *CMV::rtTA;TRE-Twist1* organoids revealed a filopodium (purple arrowhead) at the cell front surrounded by matrix clearing (B′, orange dashed lines). The TEM image is from a high-pressure frozen, freeze-substituted sample that was pre-fixed with 3% glutaraldehyde. Scale bars: 1 μm. (C-E) To test for the presence of ECM degradation, *CMV::rtTA;TRE-Twist1* organoids were cultured in Matrigel mixed with DQ-Collagen IV and labeled with CellTracker Red. Organoids were imaged by a combination of DIC and confocal microscopy to acquire both single slices and *z*-stacks. Without *Twist1* induction, organoids completed branching morphogenesis and displayed some ECM degradation at branch bifurcation points (C). With *Twist1* induction, disseminated cells were observed in the ECM with fluorescent, proteolytic trails (D-D″,E). This trail was detected to extend back to the main organoid for more proximate cells (E). Scale bars: 10 μm. (F,G) To test for the requirement for pericellular proteolysis, *CMV::rtTA;TRE-Twist1* organoids were cultured with one of two MMP inhibitors, GM6001 or Batimastat. Panels in F show representative images of organoids cultured with vehicle (DMSO) or 100 µM of inhibitor. Red arrowheads in F indicate disseminated cells. (G)The dot plot shows dissemination normalized to the median number of disseminated cells in the vehicle control. Each dot represents an organoid, pooled across three independent biological replicates. Dissemination was significantly reduced with GM6001 at 100 µM (*P=0.0351; *n=*65 organoids) and with Batimastat at 10 µM (****P<0.0001; *n=*98 organoids) and 100 µM (****P<0.0001; *n=*73 organoids) as compared to control (*n=*160 organoids). There was no significant inhibition at 10 µM GM6001 (*P*=0.1778; *n=*79 organoids). Scale bars: 50 μm. Veh, vehicle; ns, not significant.

The deformability of Twist1^+^ cells, particularly of the nucleus, was most suggestive of the amoeboid migration mode. However, amoeboid movement can describe a range of distinct motilities, including blebbing and actin polymerization-driven modes, characterized by absent or weak adhesive forces to the substrate, respectively (Friedl and Wolf, 2010; Lämmermann and Sixt, 2009). We thus sought to further characterize the phenotype of Twist1^+^ migration based on protrusive activity. We examined the dynamics of protrusions in migrating cells using the green membrane fluorescence of Twist1^+^ cells that had disseminated from genetic mosaic epithelium (*R26::LSL-rtTA;TRE-Twist1;mT/mG* mice; Fig. 5A-A′). Nascent protrusions were observed to elongate, widen, and then fully retract and collapse over a time scale of several hours (Fig. 5A,A′). Developing protrusions were often bulbous in appearance, with fine, dendritic-like branches emerging both at the tip and laterally (Fig. 5A′). When stationary, cells were observed to extend multiple protrusions in different directions, as if sampling their surroundings. When migrating, a single, primary filopodium with smaller offshoots was typically localized at the cell front, in the direction of the migration path (Fig. 5A, blue arrows). Twist1^+^ cells can thus initiate and retract multiple protrusions simultaneously, with the predominant filopodium at the leading edge.

### Twist1^+^ cell dissemination involves but does not require pericellular proteolysis

We next examined the ultrastructure of disseminated cells in the matrix. By TEM, disseminated cells had filopodia at the migrating fronts and rounded cell bodies at the rear (Fig. 5B, purple arrowhead indicates the filopodium). Similar to our analysis of protrusive cells at the tissue surface (Fig. 2D), we identified regions of decreased electron density around filopodia and membrane protrusions of disseminated cells (Fig. 5B′, orange dashed lines). We hypothesized that these areas represented proteolytic matrix clearing. However, the expectation was that amoeboid movement occurs in the absence of matrix proteolysis, which instead typifies a mesenchymal mode of migration (Lämmermann and Sixt, 2009; Wolf et al., 2003).

We therefore sought to test for the presence of pericellular proteolysis during dissemination by culturing constitutively *Twist1*-expressing organoids in Matrigel mixed with the quenched fluorescent protein substrate DQ-collagen IV. DQ-protein substrates do not fluoresce until they are cleaved by a protease, at which point they fluoresce green. Normal Twist1^–^ organoids showed ECM proteolysis restricted mostly to branch bifurcation points (Fig. 5C). Twist1^+^ organoids had ECM proteolysis both around the circumference of the main organoid and internally (Fig. 5D). Pericellular degradation was prominent around disseminated cells in the ECM, even those distant from the main organoid (Fig. 5D′,D″). A proteolytic trail could often be traced from the disseminated cell back to the main organoid (Fig. 5E), which we infer to demarcate the cell's migration path. We conclude that Twist1^+^ cells proteolytically remodel the ECM as they migrate through the matrix.

To assess the requirement for proteolysis in Twist1-induced dissemination, we utilized two broad-spectrum matrix metalloproteinase (MMP) inhibitors, GM6001 or Batimastat, at 100 nM, 1 µM, 10 µM, and 100 µM. For GM6001, there was no significant difference in the number of disseminated cells per organoid at 100 nM, 1 µM, and 10 µM. Dissemination was reduced by 33% at 100 µM GM6001 (*P=0.0351). For Batimastat, there was no significant difference at 100 nM, while dissemination was reduced by 50% at 1 µM (*P=0.0216) and by 66% at 10 µM and 100 µM (****P<0.0001) as compared to vehicle control (Fig. 5F,G). At higher concentrations, these inhibitors are not expected to be selective for MMPs and rather function to more generally block proteolysis. Given that they only partially inhibited dissemination even at the highest dose, we conclude that MMP-mediated ECM-degradation may facilitate dissemination but is not required.

### Disseminating Twist1^+^ cells demonstrate high persistence and low velocity in their initial migration path away from the epithelium

**Fig. 6.**
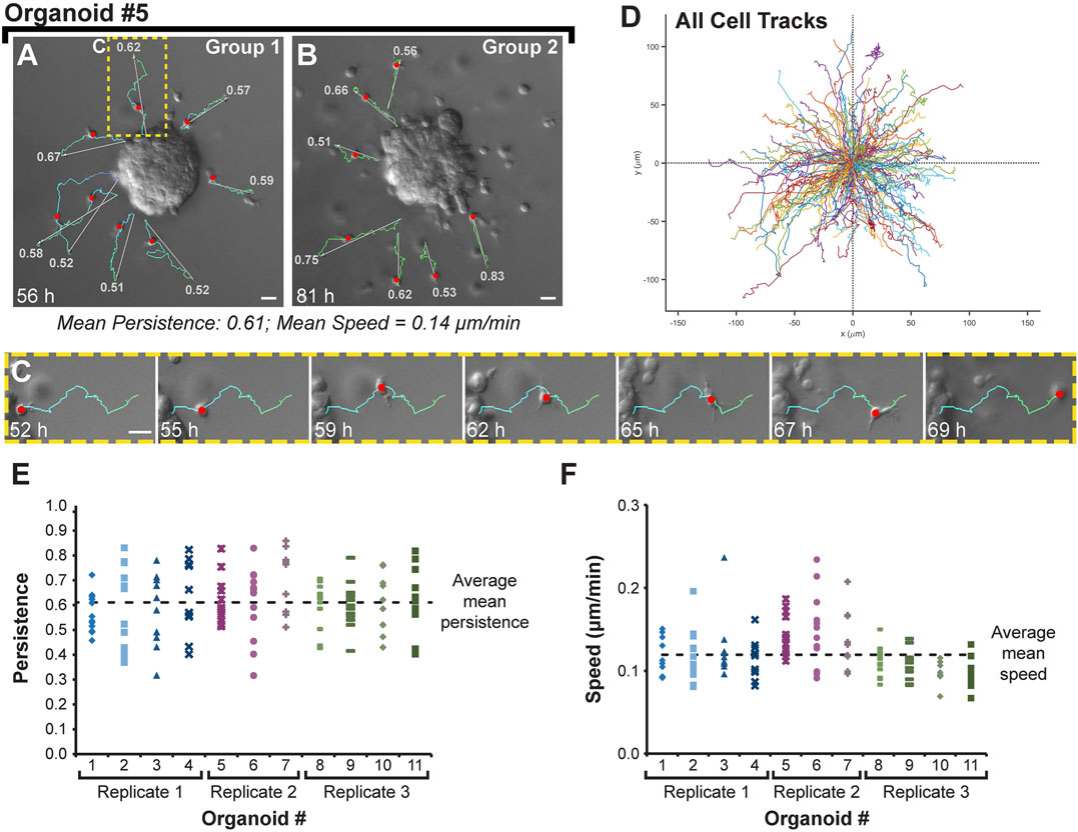
**Twist1^+^ cells disseminate with high directional persistence and slow migration speeds.** (A-C) In DIC time-lapse movies of *CMV::rtTA;TRE-Twist1* organoids, individual cells were tracked for at least 10 h following initial detachment from the basal epithelial surface. Gray arrows indicate the displacement vector and colored lines indicate the total path of the cell. Red circles indicate the position of the cell on the track at the time point of the frame. Numbered labels at the end of each track indicate the persistence. (C) A single tracked cell with persistence equal to the overall mean. Scale bars: 20 μm. (D) 133 cells were tracked across 11 movies acquired from three biological replicates. All cell tracks are plotted at a common origin. (E) Persistence values for each cell tracked are plotted within an individual movie. The horizontal dashed line (persistence=0.61) indicates the average mean persistence of migration among all sampled organoids. (F) Migration speeds (μm/min) for each cell tracked are plotted within an individual movie. The horizontal dashed line (speed=0.12 μm/min) indicates the average mean speed among all sampled organoids.

Our finding of ECM degradation around disseminated cells fit more closely within a model of mesenchymal migration. However, our time-lapse microscopy supported amoeboid cell shape changes throughout dissemination. To reconcile these observations, we measured cell speed as an independent means of distinguishing between migration modes. Amoeboid motility is characterized by high speeds (on the order of 10 μm/min), and mesenchymal motility is characterized by low speeds (0.1-1 μm/min) (Friedl and Wolf, 2010; Sahai, 2007). To determine the migration speed of Twist1^+^ cells, we tracked the migration paths of disseminating cells in DIC time-lapse movies (Fig. 6A-C shows tracks for Organoid #5). Tracking was performed from the time of initial, discernable epithelial detachment for a minimum of 10 h (mean track duration=17 h). We focused on the earliest disseminating cells to distinguish the tracked cell from nearby cells. We first noted that cells appeared to migrate radially outward away from the epithelium (Fig. 6D shows all 133 cell tracks). We calculated persistence as the displacement divided by the total track length and averaged among tracked cells for each organoid. The average mean persistence across all organoids was 0.61±0.04 (mean±s.d.) (Fig. 6E), indicating directionally persistent migration. A persistence of 0.61 can be visually interpreted in an organoid (#5) with a mean persistence equal to this value (Fig. 6A,B) and in an individual migrating cell with a persistence equal to this value (Fig. 6C). The average mean speed was 0.12±0.02 (mean±s.d.) μm/min (Fig. 6F). Combined with the presence of pericellular proteolysis, this low speed was more consistent with mesenchymal motility and suggested moderate to high adhesivity to the ECM. We conclude that disseminating Twist1^+^ cells migrate relatively slowly away from the main organoid with high directional persistence. Taken together, our data demonstrate that Twist1^+^ cells display features of both amoeboid and mesenchymal modes of migration.

### Proliferation occurs during every step of Twist1-induced dissemination

A major concept in the EMT model is that disseminating cells are growth-arrested and only reinitiate proliferation when forming a metastatic site as part of an MET (Brabletz, 2012). Whereas studies have differed on the requirement for stemness in the disseminated state, EMT-associated growth arrest has remained a common feature (Ocaña et al., 2012; Tsai et al., 2012). EMT transcription factors can inhibit proliferation, which in turn is thought to favor invasion (Peinado et al., 2007; Thiery et al., 2009; Vega et al., 2004). Another conceptual model for dissemination is the ‘go-or-grow’ hypothesis, which proposes that dissemination and proliferation are mutually exclusive (Brabletz et al., 2001; Gao et al., 2005).

**Fig. 7.**
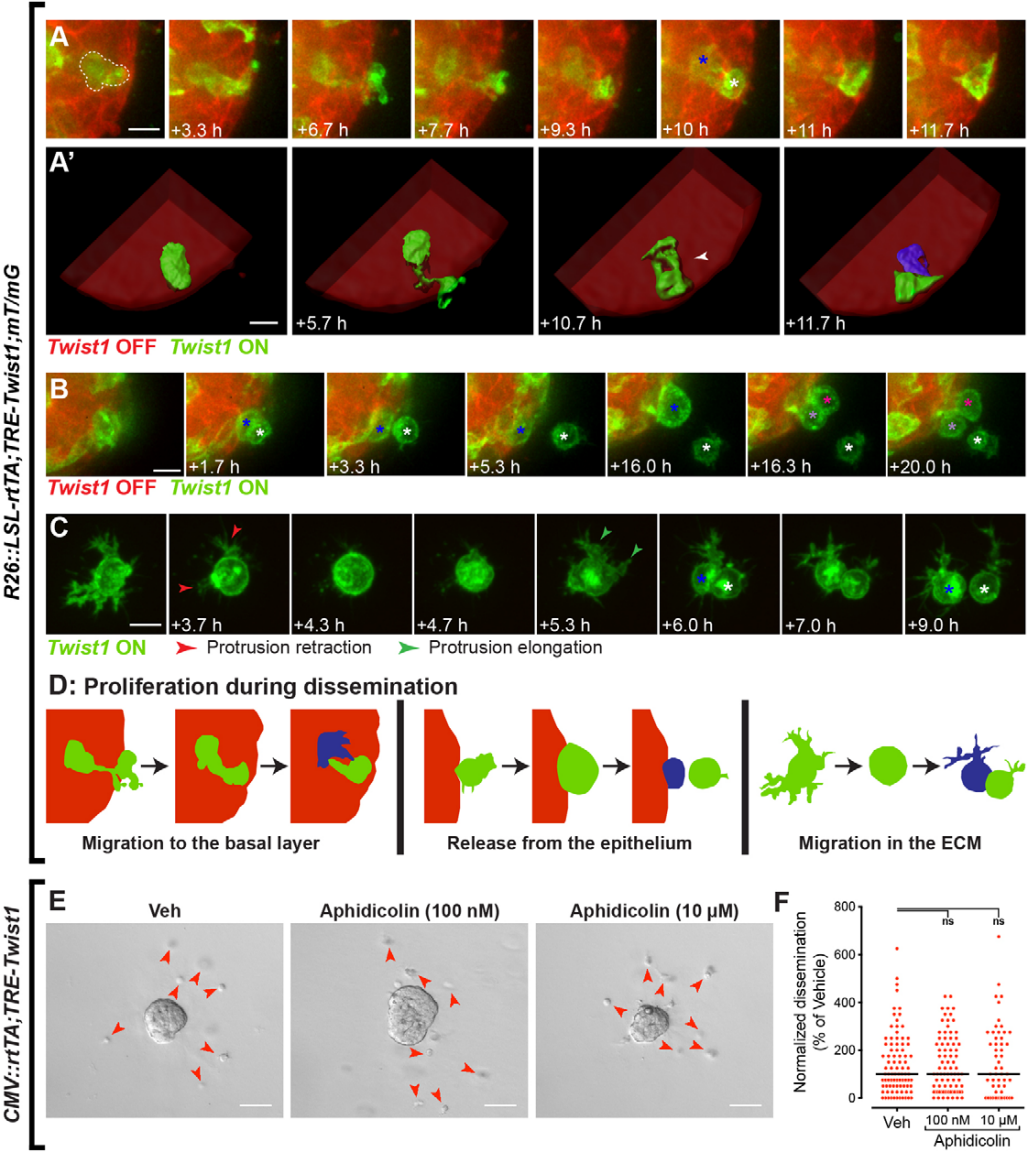
**Proliferation occurs throughout Twist1^+^ cell dissemination.** (A-D) To monitor for proliferation events during dissemination, Adeno-CMV-Cre was used to induce low-level genetic mosaic activation of *Twist1* in isolated *R26::LSL-rtTA;TRE-Twist1;mT/mG* organoids. Individual Twist1^+^ cells were tracked in real-time by confocal microscopy. Cell division was observed during basal migration (A, *n=*12 divisions in 12 organoids from three biological replicates); during cell release from the epithelium (B, *n=*6 divisions in five organoids from three biological replicates); and during migration in the ECM (C, *n=*9 divisions in six organoids from two biological replicates). White and blue asterisks in A-C denote daughter cells. Panels in A′ depict 3D reconstructions of the cell surfaces during cell division. The final positions of the daughter cells in A′ are displaced in the *z*-axis. White arrowhead in A′ indicates the point of cell division. Scale bars: 10 μm. (E,F) To test for the requirement for proliferation, *CMV::rtTA;TRE-Twist1* organoids were cultured with aphidicolin. Panels in F show representative images of organoids cultured with vehicle (DMSO), 100 nM aphidicolin, and 10 µM aphidicolin. Red arrowheads in E indicate disseminated cells. (F) The dot plot shows dissemination normalized to the median number of disseminated cells in the vehicle control. Each dot represents an organoid, pooled across three independent biological replicates. Dissemination was not significantly reduced at either 100 nM (*P*>0.9999; *n=*84 organoids) or 10 µM (*P*>0.9999; *n=*54 organoids) aphidicolin as compared to control (*n=*89 organoids). Scale bars: 50 μm. Veh, vehicle; ns, not significant.

We sought to determine whether Twist1^+^ cells could proliferate by monitoring cell division during each step of dissemination. We observed proliferation during basal migration of internal Twist1^+^ cells, which typically resulted in one daughter cell in an internal layer and the second daughter cell in the basal layer (Fig. 7A,A′). Proliferation was also observed during cell release. Cells on the tissue surface rounded up and enlarged, retracted protrusions, and divided such that one cell was released from the epithelium and the other remained attached (Fig. 7B). Finally, we detected proliferation in cells migrating in the matrix. During this process, the cell transiently stopped migrating, retracted any protrusions, and rounded up before undergoing cell division (Fig. 7C). The two daughter cells then elaborated new protrusions away from the plane of cell division and continued migrating (Fig. 7C). Taken together, these data show that proliferation can occur during every stage of dissemination (Fig. 7D). As cell proliferation events were observed at the basal surface of Twist1^+^ organoids during cell release, we next asked whether proliferation was required for dissemination. We cultured Twist1^+^ organoids in the presence of the mitosis inhibitor aphidicolin, at 100 nM, 1 µM (not shown), and 10 µM, and quantified the number of disseminated cells. Dissemination was not significantly inhibited at any of these doses as compared to vehicle control (Fig. 7E,F). We conclude that while Twist1^+^ cells do proliferate, cell division is not required for dissemination.

## DISCUSSION

**Fig. 8.**
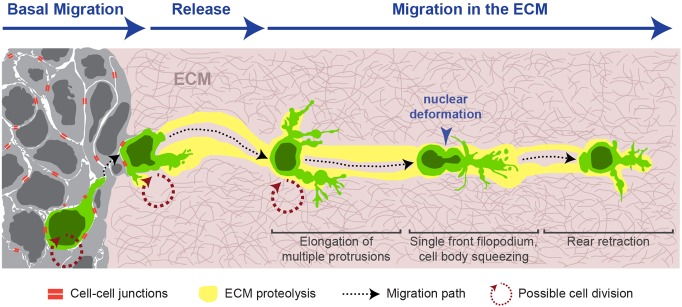
**Twist1^+^ epithelium retains intercellular junctions, and Twist1^+^ cells disseminate via a hybrid migration mode.** Our Twist1-induced dissemination assay provides a new model for how single cells disseminate from epithelial tissues. Contrary to expectation, cells within Twist1^+^ epithelium display an increased total number of junctions per cell compared to normal epithelium. Multiple, morphologically distinct categories of junctions connect interior cells and are also observed connecting cells protruding into the ECM. A single Twist1^+^ cell (green) migrates within the epithelium to the basal tissue surface, releases from the epithelium, and migrates in the ECM through cycles of amoeboid cell shape changes involving nuclear deformation and cortical constriction. At the same time, cells elaborate dynamic protrusions and degrade the surrounding ECM during migration, leaving behind a proteolytic trail. Twist1^+^ cells thus migrate with aspects of amoeboid and mesenchymal motility. Cell division can occur throughout this process.

In this study, we sought to describe the cellular basis for single-cell dissemination from epithelial tissues. We used 3D culture of *Twist1*-expressing epithelium as a model system and a combination of light and electron microscopy for analysis. Twist1, a bHLH transcription factor, is a prototypical EMT inducer and a major regulator of invasion, metastasis, cancer stemness, and tumor initiation (Beck et al., 2015; Mani et al., 2008; Schmidt et al., 2015; Tsai et al., 2012; Yang et al., 2004). The EMT model proposes that Twist1 induces dissemination by loss of cell-cell junctions, gain of mesenchymal motility, and growth arrest. In contrast, our data demonstrate that Twist1^+^ epithelium contains abundant cell-cell junctions and that disseminated cells retain the ability to adhere to normal, Twist1^–^ epithelial cells (Fig. 8). Twist1^+^ cells migrate in the ECM via a hybrid migration mode, with amoeboid morphology and pericellular proteolysis. Finally, Twist1^+^ cells can proliferate during each step of dissemination.

### Junctional adhesion

The conventional paradigm is that single-cell dissemination is induced by a loss of adhesion and cell-cell interactions. Instead, Twist1^+^ epithelium displayed an increase in cell-cell junctions, and cells retained junctions even when protruding into the ECM. Classification of these junctions based on ultrastructural morphology was challenging, as they did not have the stereotyped morphologies seen in simple polarized epithelia in other organs. We speculate that these junctions represent both desmosomes and TJs. However, we cannot rule out that they have mixed molecular character. Although TJ protein loss can contribute to tumor cell dissemination, TJ overexpression has also emerged as a driver of tumor growth and metastasis through regulation of intracellular signaling (Leech et al., 2015). Some of the electron-dense regions of close membrane apposition may also be adherens junctions (AJs), as distinguishing TJs and AJs is difficult in the mammary epithelium (Pitelka et al., 1973; Underwood et al., 2006). Given the requirement for E-cadherin in Twist1^+^ single-cell dissemination (Shamir et al., 2014) and in breast cancer metastasis to bone (Wang et al., 2015), we speculate that intercellular junctions have an underappreciated role in cancer progression. Since competing conceptual models predict either invasion promoting or suppressing roles for adhesion proteins, functional experiments will be required to elucidate the precise role of different junctional complexes across different cancers.

### Migratory modes

Classification of 3D migratory mechanisms has largely relied upon studies that start from single tumor cells or other cell types, such as leukocytes and fibroblasts, rather than from epithelial tissues. Within this framework, the transdifferentiation in EMT predicts that cells acquire mesenchymal motility through reorganization of their cortical actin cytoskeleton, elongation and formation of filopodia or invadopodia, and expression of MMPs (Lamouille et al., 2014). In contrast, our results demonstrate a migration strategy that involves simultaneous amoeboid cell shape changes and dynamic, actin-rich protrusions associated with pericellular proteolysis, phenomena typically considered mutually exclusive (Friedl and Wolf, 2010; Lämmermann and Sixt, 2009; Wolf et al., 2003). The slow migration speeds of Twist1^+^ cells suggest high adhesion to and remodeling of the ECM, consistent with the fact that Twist1 regulates genes involved in ECM composition and organization (Shamir et al., 2014). However, proteolysis was observed but not required, suggesting that amoeboid motility may be the best paradigm for Twist1^+^ cell migration. We speculate that localized protrusive activity and ECM remodeling at the cell front may together explain the highly directionally persistent migration away from the epithelium. It is worth noting that the composition, rigidity, and pore size of the ECM can regulate cell migration strategy and the requirement for proteolysis (Egeblad et al., 2010; Nguyen-Ngoc et al., 2012; Wolf et al., 2013). Therefore, it is possible that the features of Twist1-induced dissemination could differ in other ECM substrates. It would be particularly interesting to determine the behavior of Twist1^+^ cells in 3D collagen I gels with defined rigidity and pore size, as collagen I provides a more physiologic model of the interstitial matrix surrounding breast tumors.

### Tradeoff between migration and proliferation

Both EMT and the go-or-grow hypothesis propose that migration and proliferation are mutually exclusive cell states (Gao et al., 2005; Tsai et al., 2012). The EMT model further links proliferation capacity to cell fate, such that disseminated cells must revert to an epithelial cell state via a mesenchymal-epithelial transition to reinitiate proliferation (Nieto, 2013). In developmental EMT, neural crest cells are G1-arrested during delamination from the neural tube (Burstyn-Cohen and Kalcheim, 2002), and in *Caenorhabditis* *elegans* anchor cells, invasion requires G1 cell-cycle arrest for differentiation, pro-invasive gene expression, and invadopodia formation (Matus et al., 2015). We observed proliferation throughout dissemination but did not detect any consistent timing of proliferation relative to cell release, nor did inhibition of proliferation block dissemination. Our data do not exclude the possibility that downregulation of *Twist1* may promote outgrowth at the metastatic site, as this process was not modeled in our assay. It is worth noting that Twist1^+^ mammary epithelial cells survive as individual cells in the ECM without traditional pro-survival cues from adherent cell neighbors, and so Twist1 may promote cell survival in this context.

### Conclusions

In this study, we have defined the cellular and ultrastructural basis for Twist1-induced epithelial dissemination and demonstrated that it occurs despite extensive intercellular junctions and persistent adhesive capacity. Functional experiments will now be important to determine how different adhesion systems contribute to or resist dissemination across different model systems. These analyses would be greatly aided by an immuno-EM-based analysis of the molecular characteristics of the various intercellular junctions that we observe. Future studies are also needed to elucidate the molecular programs driving Twist1-induced migration and to assess the impact of heterotypic intercellular interactions on invasion and dissemination.

## MATERIALS AND METHODS

### Mouse strains

The *CMV::rtTA* transgenic line was a kind gift of Feng Cong and Harold Varmus (National Cancer Institute, Bethesda, MD). The *K14::Actin-GFP* transgenic line (Vaezi et al., 2002) was a kind gift of Elaine Fuchs (The Rockefeller University, New York, NY). The *Twist1-tetO_7_-luc* (*TRE-Twist1*) transgenic line was previously described (Tran et al., 2012). *mT/mG* (Muzumdar et al., 2007) and *R26::Lox-Stop-Lox-rtTA-IRES-EGFP* (*R26::LSL-rtTA*) (Belteki et al., 2005) mouse lines were acquired from the Jackson Laboratory. Mammary glands were isolated from female mice between the ages of 6-15 weeks. Mouse husbandry and procedures were all conducted under an IACUC-approved animal protocol.

### Isolation and 3D culture of primary mammary epithelial organoids

We used a combination of mechanical disruption, collagenase/trypsin digestion, and differential centrifugation to purify fragments of primary mammary epithelial ducts, termed ‘organoids’, as previously described (Nguyen-Ngoc et al., 2015; Shamir et al., 2014). Organoids were embedded in 3D Matrigel (354230; Corning) at 2-3 organoids/μl and plated as 100 μl suspensions in 24-well coverslip-bottomed plates (662892; Greiner Bio-One) over a 37°C heating block. Gels were allowed to polymerize for 30-60 min at 37°C and then cultured in organoid medium: DMEM (D6546; Sigma) with 1% insulin-transferrin-selenium (51500-056; GIBCO) and 1% penicillin-streptomycin (P4333; Sigma). The following day, organoid medium was supplemented with 5 μg/ml doxycycline (Shanghai RenYoung Pharmaceutical Co., Ltd) to induce *Twist1* expression. Optionally, 2.5 nM FGF2 (F0291; Sigma) was also added to induce branching morphogenesis. Due the lability of doxycycline, medium was replaced every 48 h, including in control samples, for the duration of culture.

### Adenoviral delivery of Cre recombinase

Isolated *R26::LSL-rtTA;TRE-Twist1;mT/mG* organoids were infected with Adeno-CMV-Cre (1045; Vector Biolabs) to induce recombination and *rtTA* expression. Infections were conducted by adding 1 μl Adeno-CMV-Cre to 1000 organoids in 50 μl of DMEM to yield recombination in 50-75% of cells (approximately 10^4^ PFU per organoid). To induce lower levels of recombination, Adeno-CMV-Cre was first diluted 1:10 or 1:20 in DMEM and 1 μl added to the organoid suspension. Organoids were incubated for 1-2 h at 37°C, washed once with DMEM, and embedded in Matrigel.

### Confocal microscopy

Confocal imaging was performed on a spinning-disk confocal microscope (Solamere Technology Group) with an XR/MEGA-10 S30 camera (Stanford Photonics, Inc.), as previously described (Ewald, 2013; Ewald et al., 2011). An LD C-Apochromat 40×/1.1 W Korr objective lens (Carl Zeiss) was used for high magnification single and time-lapse image acquisition, with water and oil used as the imaging media, respectively. Acquisition of both fixed and time-lapse images was performed using a combination of μManager (Edelstein et al., 2010) and Piper (Stanford Photonics, Inc.). Imaris (Bitplane) was used to analyze time-lapse movies, perform surface rendering, place scale bars, and export individual TIFFs. Adobe Photoshop was used as needed to adjust levels for each channel across entire images to maximize image clarity.

### Differential interference contrast (DIC) microscopy

Time-lapse imaging of mammary organoids was conducted using an LD Plan-Neofluar 20×/0.4 Korr Ph2 objective lens and a Cell Observer system with an AxioObserver Z1 and an AxioCam MRM camera (Carl Zeiss). Images were acquired at 20-min intervals for 5-7 days. Temperature was maintained at 37°C and CO_2_ at 5%. AxioVision (Carl Zeiss) was used to analyze both fixed images and time-lapse movies, place scale bars, and export individual TIFFs. Adobe Photoshop was used to adjust levels on entire images to maximize image clarity.

### Glycosaminoglycan staining with Ruthenium Red

We isolated epithelium from a *CMV::rtTA;TRE-Twist1* mouse and cultured organoids for 5 days in Matrigel in organoid medium supplemented with 2.5 nM FGF2. *Twist1* was induced in half of the organoids with 5 μg/ml doxycycline. Embedded organoids were then fixed in 3% glutaraldehyde to preserve for shipping to Lawrence Berkeley National Laboratory. There, samples were high-pressure frozen either unstained or stained using Luft's Ruthenium Red (RR) method (Luft, 1971) in combination with microwave-assisted processing. All microwaving procedures were carried out using a Pelco Biowave microwave oven with a Pelco ColdSpot insert cooled by a Pelco SteadyTemp chilled cooling system (Ted Pella Inc., Redding, CA). Briefly, samples were microwaved in 0.05% RR in 0.1 M sodium cacodylate buffer at 150 W for 1-min-ON, 1-min-OFF, 1-min-ON and rinsed three times by microwaving in fresh buffer for 40 s at 150 W. Samples were then microwaved in 0.05% RR and 1% osmium tetroxide for 1-min-ON, 1-min-OFF, 1-min-ON at 150 W and rinsed three times by microwaving in fresh buffer for 40 s at 150 W. Samples were stored in 0.1 M sodium cacodylate buffer at 4°C until high-pressure freezing.

### High-pressure freezing, freeze substitution, and resin embedding

Both unstained and RR-stained samples were placed in 1-mm-wide by 200-μm-deep aluminum freezing hats and, before freezing, were surrounded with 20% BSA, used as a cryoprotectant. Samples were then cryoimmobilized using a high-pressure freezer (HPM-010; Bal-tec, Inc.) and freeze-substituted in 1% osmium tetroxide and 0.1% uranyl acetate in acetone, as previously described (McDonald and Webb, 2011). Upon completion of freeze substitution, samples were progressively infiltrated with an epon-araldite resin using a quick infiltration procedure, as previously described (McDonald, 2014). Polymerization in epon-araldite resin was performed by flat embedding between two glass slides at 60°C overnight to allow for precise localization of features of interest (Müller-Reichert et al., 2003).

### TEM

Samples were sectioned into 70-90-nm-thin sections using an Ultramicrotome (UC6; Leica). Sections were then collected onto formvar-coated, rhodium-enforced copper 2-mm slot grids (M2010-CR; Electron Microscopy Sciences). The grids were post-stained with 2% uranyl acetate followed by Reynold's lead citrate, for 5 min each. The sections were imaged using a Tecnai 12 TEM (FEI), operated between 480× and 18,500× at 120 kV under normal conditions. Images were recorded using an Orius SC1000B CCD with Digital Micrograph 3 software (Gatan Inc.).

### Montaging TEM images

SerialEM software was used to collect wide-field montages for overview imaging of complete organoid cross-sections, as well as for high-magnification imaging of large regions of interest containing multicellular features (Mastronarde, 2005). The mosaic of images obtained by SerialEM was reconstructed using the *blendmont* utility in the IMOD software package, which aligns the smaller images and blends overlapping edges (Kremer et al., 1996). ImageJ software (Abramoff et al., 2004) and Adobe Photoshop were used to crop images, place scale bars, and adjust brightness and contrast across entire images, as needed.

### Quantification of junctions

TEM montages of three Twist1^+^ and three Twist1^–^ organoids were selected for analysis of junctional adhesion among internal cells. For each organoid, we labeled and counted all cells that were at least one cell layer interior from the ECM. We excluded cells lining a lumen (determined by visible microvilli and tight junctions) or other internal cavities, cells without a visible nucleus (to define a minimum cell cross section for analysis), cells in which cell-cell borders were difficult to delineate or were obscured by an imaging artifact, and cells that were cut off at the edge of the montage. From the population of interest, we used a pseudorandom number generator to select 10 cells for quantification from each organoid. There were 140 total eligible cells for Twist1^+^ epithelium and 150 total eligible cells for control. Adobe Illustrator CS6 was used to annotate the cells for junctions according to the following criteria. Bar junctions were of variable length, contained no detectable intercellular space between adjoining cell membranes, and localized a varying accumulation of electron density at the membrane. Punctate junctions had limited lateral extent along the membrane and localized electron density in the cytoplasm beneath the cell-cell contact. Sandwich junctions were of variable length, had detectable intercellular space containing varying electron density, and localized membrane density but not cytoplasmic density. Contact junctions displayed increased electron density at a single point of contact between a membrane protrusion of one cell and the main cell membrane of an adjacent cell. A bar junction was scored if the lateral edge of a membrane protrusion made contact with an adjacent cell and displayed increased electron density and no intercellular space. We excluded junctions between membrane protrusions on adjoining cells. Regions of membrane apposition, in which a cell-cell border lacked intercellular space but was difficult to resolve and did not localize electron density, were not scored as junctions. Junctions were double-counted if present in two adjoining cells used for the quantification. The number of junctions per cell in a particular class as well as the total number of junctions per cell were calculated across the 30 Twist1^+^ cells and 30 Twist1^–^ cells and statistically compared within each category using a two-tailed *t*-test with unequal variance.

### Immunofluorescence

Organoids grown in Matrigel were fixed in 4% paraformaldehyde for 10 min, rinsed three times in PBS for 10 min, embedded in Optimal Cutting Temperature compound (OCT), and frozen at −80°C. OCT blocks were sectioned at 50-μm thickness by cryostat at −20°C. Sections were placed on Superfrost Plus Gold microscope slides (15-188-48; Fisherbrand) and stored at −80°C. For antibody staining, samples were thawed at room temperature, rinsed twice in PBS for 10 min to remove OCT, permeabilized with 0.5% Triton X-100 for 1 h, and rinsed twice in PBS for 10 min. Samples were blocked for 1-3 h with 10% FBS/1% BSA, incubated with primary antibodies overnight at 4°C in 1% FBS/1% BSA, and rinsed three times in 1% FBS/1% BSA for 15 min. Incubation with secondary antibodies was conducted in 1% FBS/1% BSA overnight at 4°C. Slides were rinsed three times in PBS for 10 min, mounted with Fluoromount (F4680; Sigma-Aldrich), and sealed with coverslips. Primary antibodies used were mouse anti-Twist1 (1:50; sc-81417; Santa Cruz Biotechnology Inc.) and mouse anti-smooth muscle α-actin (1:250; A5228; Sigma-Aldrich). Secondary antibodies used were all Alexa Fluor-conjugated antibodies (1:200; Invitrogen).

### DQ Collagen

Organoids were isolated from *CMV::rtTA;TRE-Twist1* mice and embedded in Matrigel mixed with 25 μg/ml DQ-Collagen, type IV (D12052; Thermo Fisher Scientific). Organoids were cultured in organoid medium with 2.5 nM FGF2, and *Twist1* expression was induced in half of the samples with 5 μg/ml doxycycline. On day 5-7 in culture, organoids were labeled with CellTracker Red (C34552; Thermo Fisher Scientific). One 50 μg vial of CellTracker was resuspended in 73 μl sterile DMSO to make a 1 mM stock. The vial was warmed for several minutes at 37°C to dissolve the solution. CellTracker was added to organoid medium at 1 μM. Samples were stained for either 2 h or overnight at 37°C. The CellTracker-containing medium was then removed, and wells were rinsed with organoid medium with or without doxycycline two times for 20 min at 37°C. Proteolytic activity was detected as green fluorescence resulting from enzymatic cleavage of the quenched substrates. Dual DIC and confocal imaging was conducted on days 6-8 in culture with a Zeiss LSM 780 confocal microscope with a 40× LDLCI C-Apochromat objective using ZEN imaging software. Organoids isolated from the same mouse cultured in Matrigel without DQ-Collagen IV served as a negative control. We performed four biologically independent replicates and imaged at least five organoids per condition per mouse.

### Dissemination inhibition assay

Isolated organoids from *CMV::rtTA;TRE-Twist1* mice were embedded in Matrigel at a density of 1 organoid/μl and cultured in organoid medium overnight. The following day, the culture medium was replaced with medium containing 2.5 nM FGF2, 5 μg/ml doxycycline to induce *Twist1*, and either pharmacological inhibitors or vehicle (DMSO) to assay for an effect on dissemination. Medium was replaced every 48 h. Cultures were maintained for 7 days, then fixed in 4% (m/v) paraformaldehyde (in PBS with Ca^2+^ and Mg^2+^) and imaged by DIC microscopy. Dissemination was quantified as the number of disseminated cells per organoid. Dissemination did not follow a normal distribution in any of the tested conditions (D'Agostino and Pearson omnibus normality test; *P*<0.05). Therefore, dissemination was normalized intra-experimentally to the median of dissemination in the vehicle control. Normalized data from three independent biological replicates were pooled, and statistical significance was determined using the Kruskal–Wallis test with Dunn's multiple comparisons test (non-parametric, non-paired comparisons). *P<0.05; **P<0.01; ***P<0.001; ****P<0.0001.

### Cell tracking

Imaris (Bitplane) was used to perform tracking on at least 10 cells per movie in a total of 11 movies of *CMV::rtTA;TRE-Twist1* organoids across three biological replicates. All organoids were cultured in organoid medium with 2.5 nM FGF2 and 5 μg/ml doxycycline. Tracks were generated for cells that could be followed from initial detachment over at least 10 h. The center of the main cell body was used as the reference point across frames. Cells were tracked until they were no longer visible (e.g. migrated out of focus) or alternatively began to divide or form a secondary site. Cells were excluded if they significantly interacted with surrounding disseminated cells. Persistence was calculated as the displacement divided by the total track length and averaged across all cells within a single movie. Migration speed was calculated as the total track length divided by the track duration and averaged across all cells within a single movie.

### Image segmentation

The segmentation in Fig. 3C, Fig. 4D-F, Fig. 7D, and Fig. S1A was manually performed in Adobe Illustrator. From the multi-channel images, individual channels (displayed in black and white) were transferred from Adobe Photoshop, and the pen tool was used to trace the fluorescence signal. The contrast correction used was the same as for the final, multi-channel image displayed. No additional amplification or gamma correction was used to modify the signal. Many of the small protrusions were much dimmer than the main cell membrane and were more visible in the black and white, single channel panels than in the green/red overlay. The 3D reconstruction in Fig. 7A and Fig. S1B was performed in Imaris by using the surface-rendering tool. The green membrane fluorescence was used to generate the surface of the cell(s) of interest, and the red membrane fluorescence was used to generate a volume for the organoid. Finally, the image was rotated in ‘Surpass’ mode to change the viewing angle.
